# Recall of patients on community treatment orders over three years in the OCTET CTO cohort

**DOI:** 10.1186/s12888-016-1102-4

**Published:** 2016-11-09

**Authors:** Jorun Rugkåsa, Ksenija Yeeles, Constantinos Koshiaris, Tom Burns

**Affiliations:** 1 Health Services Research Unit, Akershus University Hospital, 1478 Lørenskog, Norway; 2 Department of Psychiatry, University of Oxford, Oxford, UK; 3 Department of Primary Care, University of Oxford, Oxford, UK

**Keywords:** Community Treatment Order, Community compulsion, Recall, Revocation, Outpatient Commitment, OCTET trial

## Abstract

**Background:**

Randomised studies consistently show that Community Treatment Orders (CTOs) do not have the intended effect of preventing relapse and readmissions of patients with severe and enduring mental illness. Critics suggest this in part can be explained by RCTs studying newly introduced CTO regimes and that patients therefore were not brought back to hospital for short-term observations (‘recall’) as frequently as intended. Our purpose was (i) to test the hypothesis that CTO practice as regards recall of patients to hospital in England and Wales was as rigorous under the OCTET trial period as in current routine use and (ii) to investigate the reasons for and outcomes of recalls and whether this changed over time.

**Method:**

Thirty six-month observational prospective study of 198 patients in the OCTET Follow-up Study.

**Results:**

Forty percent of patients were recalled, 19 % more than once. This is in line with current national use. Deterioration in clinical condition was the most common reason for recalls (49 %), and 68 % of recalls resulted in revocation of the order (i.e., retention in hospital under compulsion). This pattern remained stable over time.

**Conclusion:**

The use of recall cannot explain why RCTs have not confirmed any benefits from CTOs, and their continued use should be reconsidered.

**Trial registration:**

The OCTET Trial was retrospectively registered on 12 November 2009 (ISRCTN73110773)

## Background

Deinstitutionalisation of mental health services has resulted in severely ill patients increasingly being treated in the community. In response, legal regimes for involuntary out-patient treatment (Community Treatment Orders, CTOs) have been developed. First introduced in North America and Australasia, they are also becoming a feature of mental health legislations in much of Europe and are now available in more than 75 jurisdictions worldwide [1]. CTOs permits compulsory treatment outside of hospital. They target patients deemed to need supervision but who are well enough for this to take place outside hospital. The aim is to break the pattern of repeated admissions by facilitating a period of stability so that the patient settles in the community and eventually returns to voluntary care.

CTOs were introduced into the amended Mental Health Act for England and Wales (MHA) in November 2008 [2]. The legal criteria include that the patient suffers from a mental disorder for which they require treatment to protect their health and safety or that of others; that the treatment (which must be available) can continue in the community, and; that it is necessary for the responsible clinician to be able to exercise the power of rapid recall to hospital for observation [2]. Recalls may take place when patients show signs of deterioration or they breach one of the two mandatory conditions of making themselves available for assessment. A recall to hospital for observation or treatment can last for up to 72 h after which there are three options. The order can be revoked and the patient remains in hospital for involuntary treatment; they return to the community on the CTO, or; they are discharged from involuntary treatment altogether. The recall mechanism is thus central to how CTOs were envisaged to work. Similar mechanisms exist in other CTO regimes [3].

Current evidence shows that CTOs are not effective in their stated purpose of reducing relapse and readmission [4–6]. Around 35 outcome studies and three RCTs have investigated the effect on hospitalisation (rates, duration and time to readmission following placement on CTO) and community service usage. While non-randomised studies vary in quality and have inconsistent findings, all analyses of randomised data found no effect of CTOs [4, 7–9]. A meta-analysis of the patients from the three RCTs also found no effect on admissions or bed days or any improvement in psychiatric symptoms or global functioning [7]. Additionally, the OCTET trial testing the English regime [10] found no effect on subgroups [11] or any long-term (36 months) effect on hospital outcomes or on engagement with services [12].

One explanation advanced for the lack of observed positive effects of CTOs is that newly introduced regimes are not implemented as rigorously as intended [4, 13, 14], although there is little published data to support this proposal. Specifically it is suggested that clinicians were inexperienced in the use of CTOs when the studies were conducted and that recall to hospital was not used as often as intended, preventing patient improvement [14, 15]. There is very little evidence on which to base such claims of inadequate recalls because this data is not reported in effectiveness studies. National prevalence data in England and Wales show that on 31 March 2015, 5461 people were subject to a CTO. No data is available on how long these patients had been on CTO. In the preceding year, available incidence figures show that 2369 recalls and 1427 revocations were made. This would suggest that around 43 % of CTO patients in that period were recalled, but some may have been recalled more than once so the true figure is probably lower. Of these recalls, 60 % ended in revocation. These figures have remained stable over the last 5 years [16].

### Aims of the study

To address the concern over the validity of the evidence base, we tested the hypothesis that early implementation of CTOs in England and Wales was equally rigorous in terms of recalling patients as is the case routinely now 8 years after their introduction. We used data from the three year follow-up of the OCTET cohort [12]. A secondary aim was to examine the reasons for and outcomes of these recalls, and whether this changed over time.

## Methods

The OCTET trial recruited patients in 32 National Health Service (NHS) hospitals across England and followed them up for 12 months. At randomisation, all were in involuntary hospital treatment, aged 18–65 years, with a psychosis diagnosis and considered to need CTO on discharge. Patients were randomly allocated (50:50) to leave hospital on a CTO or to voluntary status via Section 17 leave of absence [10]. The OCTET Follow-Up Study then followed the cohort of 333 patients for a further 24 months (i.e., 36 months, 1095 days, in total) [12]. For the present observational prospective cohort study we selected those patients from the OCTET Follow-up Study who at any time during the follow-up period were on a CTO. We applied no further inclusion or exclusion criteria.

For each patient we used data from the date of first placement on CTO until 36-months after randomisation in the original trial. Data were collected, by independent researchers, from NHS medical records, including trusts’ Mental Health Act Offices, in the period from November 2008 to February 2014. Socio-demographic and clinical characteristics were collected through patient interviews at baseline [10, 12].

The number of occurrences of recalls was counted. For each recall we classified the recorded reasons in pre-defined categories (deterioration in health; medication non-compliance; disengagement), and recorded their outcomes (continued CTO; revocation; discharge to voluntary care).

To allow investigation of whether practice changed over time, either with experience of individual patients or with increased familiarity with the CTO regime, we used two separate methods. First, we examined the reason for and outcome of patients’ first, second, third and fourth recall. Second, we divided the data on patients’ first ever recalls in two time periods: period 1, the 0–12 months of a patient’s participation in the study (0–365 days) and period 2, 13–36 months in the study (366–1095 days).

Global functioning and severity of symptoms were assessed at baseline, using the Global Assessment of Functioning (GAF) [17] and the Brief Psychiatric Rating Scale (BPRS) [18] respectively.

We report the number and percentage of observed values for binary and categorical variables and, depending on data distribution, mean with standard deviation (SD) or median with interquartile range (IQR) for continuous variables. The descriptive statistics are provided for the whole sample and by time period.

## Results

### Sample and baseline characteristics

Of the 333 patients in the OCTET Follow-up study, 198 were subject to a CTO during the 36 months and so included in this analysis. Socio-demographic and clinical characteristics of the sample at baseline are displayed in Table 1.

**Table 1:** Patients characteristics at baseline (*n* = 198)

	Missing data		n/%, Mean (SD) Median [IQR]	
	n	%		
Demographics				
Age (years)	0	0 %	39.6	(11.2)
Male	0	0 %	135	68 %
Years of education	3	2 %	11.9	(1.7)
Ethnicity	0	0 %	-	-
White	-	-	114	58 %
Black	-	-	51	26 %
Asian	-	-	19	10 %
Mixed and other	-	-	14	7 %
Born in UK	0	0 %	155	78 %
Social/living situation				
Married/co-habiting	1	1 %	16	8 %
Patients with identified carer	20	10 %	72	36 %
Independent accommodation	2	1 %	147	74 %
Living alone/homeless	12	6 %	143	72 %
Clinical status				
Schizophrenia	0	0 %	166	84 %
Brief Psychiatric Rating Scale (BPRS)	14	7 %	39	[33, 45]
Global Assessment of Functioning (GAF)	15	8 %	38.5	[31, 48.5]
Clinical history				
Duration of illness (years)	4	2 %	12	[6,20]
Patients with duration of illness less than 2 years	0	0 %	7	4 %
Number of past hospital admissions	11	6 %	6	[3, 9]
Months of past hospital stay	29	15 %	16	[8, 28]
Number of past involuntary hospital admissions	19	10 %	4	[2, 7]
Criminal history				
Convictions	15	8 %	83	42 %
Imprisonment	13	7 %	55	28 %

*CTO* = community treatment orders

- = not applicable

Patients were predominantly male (68 %, 135/198) and the mean age was 39.6 (SD 11.2). They had a mean of 11.9 (SD 1.7) years of education (12 years of education is mandatory in the UK). Over half were White (58 %, 114/198), a quarter were Black (26 %, 51/198) and the remainder were Asian (10 %, 19/198) or of mixed or ‘other’ ethnic origin (7 %, 14/198). The majority of patients suffered from schizophrenia (84 %, 166/198) and the median duration of illness was 12 years (IQR 6, 20). The median level of symptoms, measured by the BPRS was 39 (IQR 33,45) and the median level of functioning as measured by the GAF was 38.5 (IQR 31,48.5), indicating a severely ill group of people. They had a median of 6 (IQR 3, 9) previous hospital admissions. Almost half the sample (42 %, 83/198) had criminal convictions and a third (28 %, 55/198) had been imprisoned.

### Rate of recall to hospital

As shown in Table 2, a total of 136 recalls were made, around one for every two CTOs. 40 % of the patients (78/198) were recalled, and 19 % (37/198) were recalled twice. 8 % (15/198) were recalled three times and 3 % patients (6/198) had four to six recalls.

**Table 2:** Reasons for and outcomes of recalls of 78 patients over 36 months by CTO and by time period

	All recalls		1^st^ recall		2^nd^ recall		3^rd^ recall		4^th^ – 6^th^ recall		1^st^ recall			
											0^th^–12^th^ month		13^th^–36^th^ month	
	(*N* = 136)		(*N* = 78)		(*N* = 37)		(*N* = 15)		(*N* = 6)		(*N* = 45)		(*N* = 33)	
	n	%	n	%	n	%	n	%	n	%	n	%	n	%
Reasons for recall														
Not recorded	29	21 %	26	33 %	1	3 %	2	13 %	0	0 %	24	53 %	2	6 %
Deterioration of health	67	49 %	36	46 %	22	59 %	7	47 %	2	33 %	11	24 %	25	76 %
Medication non-compliance	26	19 %	10	13 %	8	22 %	6	40 %	2	33 %	6	13 %	4	12 %
Disengagement	11	8 %	4	5 %	5	14 %	0	0 %	2	33 %	2	4 %	2	6 %
Other	3	2 %	2	3 %	1	3 %	0	0 %	0	0 %	2	4 %	0	0 %
Recall outcomes														
Revoked (to sec. 3, inpatient)	92	68 %	53	68 %	27	73 %	10	67 %	2	33 %	29	64 %	24	73 %
Continued CTO	42	31 %	24	30 %	9	24 %	5	33 %	4	67 %	15	33 %	9	27 %
Discharged to voluntary care	2	1 %	1	1 %	1	3 %	0	0 %	0	0 %	1	2 %	0	0 %

*CTO* Community Treatment Order

### Reasons for and outcomes of recalls

The reasons for and outcomes of all recalls are presented in Table 2, which also presents this information on successive recalls and on first recalls in periods 1 and 2.

Deterioration of health was the recorded reason for half of all recalls (49 %, 67/136). Non-compliance with medication was the reason for 19 % of recalls (26/136) and disengagement in 8 % of cases (11/136). There was no reason recorded for 33 % (26/78) of first recalls.

Deterioration of health applied to 59 % (22/37) of patients experiencing a second recall. For those patients who had their first recall in period 2, this was even higher, with 76 % (25/33) compared with 24 % (11/45) of those recalled in period 1. Non-compliance as a reason remained stable for first recalls over time periods, but there was an increase in non-compliance as the recorded reason for recalling patients for the second or third time. Non-recording decreased with subsequent recalls and also between period 1 and 2 from 53 % (24/45) to 6 % (2/33).

Sixty eight percent of all recalls (92/136) ended in revocation and the patient returning to involuntary hospital treatment. Patients remained on CTO after 31 % of all recalls (42/136). Only 1 % of recalls (2/136) ended in discharge from involuntary treatment. Fewer recalls ended in revocation in period 1 (64 %, 29/45) than in period 2 (73 %, 24/33).

## Discussion

Our sample consisted of severely ill psychosis patients with long histories of hospital admissions. The characteristics of the sample are similar to those reported in national and international studies [4, 5, 19–22].

We found that 40 % of CTO patients were recalled to hospital at least once and 19 % more than once. As we have previously reported, the 198 patients stayed on CTO for one year on average (median 346 days, IQR 180–724) [12], which means our recall rate of 40 % is broadly comparable with the current national rate of around 43 % over 12 months [16]. This supports our hypothesis that the use of recall to hospital during the OCTET trial was as rigorous as in the current, mature system.

These findings are in line with local studies: A 12 month follow-up of 65 CTOs in North England reported a 40 % recall rate over 12 months [21] and an audit of 50 CTOs in North Wales reported a 34 % recall rate [23]. These studies reported very different rates of revocation, however, 96 % [21] and 34 % [23] respectively. Two 6-month follow-ups (of 104 and 67 patients respectively) saw lower recall rates of 19 % [19] and 13 % [24], possibly due to the short time frame. They also reported high revocation rates of 70 % [19] and 100 % [24]. Nationally, 60 % of recalls ended in revocation during 2014/15 [16]; our rate of 68 % was slightly higher. There is a paucity of international studies of recall and revocation, but one small study from Victoria, Australia report a 62 % revocation level [25].

Half of all recalls in our study (49 %) were due to deterioration in the patients’ mental state and a fifth due to medication non-compliance. There was a large increase from period 1 to period 2 in clinical deterioration being recorded as the reason for first recalls. This could signify a change in use. However as the number of recalls with no recorded reason decreased from 53 % to 6 % it is more likely that this reflects improved record keeping. The majority of recalls (68 %) ended in revocation, and there was a slight increase over time. A third of recalls resulted in the patient returning to the community on the CTO. Only two recalls (1 %) resulted in discharge from compulsion. Overall, these findings suggest recalls were used as intended to intervene during relapse.

There has been some concern over clinicians’ understanding of when to use recall [26, 27], but even so, both our study and national figures suggest their use has remained stable over time. Our findings indicate that decisions to recall patients are based on judgements of the patient’s clinical state. This is also how psychiatrists explain their use of CTOs [28, 29], and it is in line with current legal understanding of how recall may be used [26].

### Strength and weaknesses

This descriptive analysis is the first to investigate CTO recalls over a prolonged period of 36 months. A key strength is the degree of data completeness for a group of severely ill patients [12]. They were recruited from 32 different NHS hospitals, covering both urban and rural areas [10]. Information on the reasons for recall was not comprehensively recorded in patient records early in the process. Percentages calculated from small sub-samples should be interpreted with caution. Our sample consists of patients recruited to the OCTET trial, which means it is restricted to those deemed suitable for CTO by clinicians who were in equipoise about the benefit of CTO vs. the control condition.

## Conclusions

Our findings support our hypothesis that the use of recall of CTO patients to hospital over the course of the OCTET RCT was in line with mature practice nationally. We have also shown that the use of recall, its reasons and outcomes did not change over time, contrary to what has been suggested as explanations for the absence of positive effects of CTOs in RCTs. There is a lack of published research on the rates, reasons and outcomes of short-term recalls of patients to hospital as part of the CTO process, and this should be a focus for future research, particularly to facilitate international comparisons.

Our findings lend support to the conclusions of existing RCTs by demonstrating that the CTOs in the OCTET trial were implemented in line with routine practice. This strengthens the evidence that CTOs do not achieve their stated aims of improving hospital outcomes and that their use should be urgently reconsidered.

## Abbreviations

BPRS: Brief Psychiatric Rating Scale
CTO: Community Treatment Order
GAF: Global Assessment of Functioning
IQR: Interquartile range
MHA: Mental Health Act
NHS: National Health Service
OCTET: Oxford Community Treatment Order Evaluation Trial
RCT: Randomised controlled trial
SD: Standard deviation
